# Vitamin D Enhances Alveolar Development in Antenatal Lipopolysaccharide-Treated Rats through the Suppression of Interferon-γ Production

**DOI:** 10.3389/fimmu.2017.01923

**Published:** 2018-01-05

**Authors:** Chengbo Liu, Ze Chen, Wen Li, Lisu Huang, Yongjun Zhang

**Affiliations:** ^1^Department of Neonatology, Xinhua Hospital, Shanghai Jiaotong University School of Medicine, Shanghai, China; ^2^MOE and Shanghai Key Laboratory of Children’s Environmental Health, Shanghai, China

**Keywords:** bronchopulmonary dysplasia, vitamin D, interferon-γ, methylation, inflammation

## Abstract

Bronchopulmonary dysplasia (BPD) is characterized by the premature arrest of alveolar development. Antenatal exposure to inflammation inhibits lung morphogenesis, thereby increasing the risk for the development of BPD. Here, we investigated whether vitamin D (VitD) enhances alveolar development in antenatal lipopolysaccharide (LPS)-treated rats, which is a model for BPD. We used an established animal model of BPD, and random assignment to the control group, LPS group, or LPS with VitD group. Levels of interferon (IFN)-γ and interleukin-4 were detected by real-time polymerase chain reaction (PCR) and enzyme-linked immunosorbent assay. IFN-γ producing CD8+ T cells were assessed by flow cytometry, and the methylation status of the VitD-response element (VDRE) was analyzed by bisulfite sequencing PCR. 25-hydroxyvitamin D levels were measured by liquid chromatography tandem mass spectrometry in maternal serum samples collected from 86 pregnant women in a prospective birth cohort enrolled from 2012 to 2013. Our results showed that VitD effectively alleviated the simplification of the lung alveolar structure in BPD rats and suppressed LPS-induced IFN-γ expression in the lung and spleen tissues. Further investigation revealed that VitD suppressed IFN-γ production in CD8+ T cells. Specifically, VitD increased the methylation percentage of the VDRE in the IFN-γ-promoter region and suppressed LPS-induced expression of IFN-γ. Additionally, we observed an association between maternal VitD exposure during pregnancy and neonatal IFN-γ levels in a prospective birth cohort, with a trend similar to that observed in the animal model. Our data suggested that supplementation of VitD could suppress IFN-γ production, resulting in improved alveolar development in an LPS-induced BPD rat model.

## Introduction

Bronchopulmonary dysplasia (BPD) has a complex pathogenesis involving multiple insults to the immature lungs that result in varying degrees of alveolar simplification and structural and functional modifications of the arterioles and bronchioles (1, 2).

The cellular and molecular mechanisms leading to abnormal lung development in BPD are poorly understood. Triggered in the immature lung by infectious complications, oxygen toxicity, mechanical ventilation, a sustained inflammatory response, and extensive remodeling of the extracellular matrix, as well as altered growth factor signaling, characterize the disease (3, 4). Several animal models of neonatal lung injury or BPD with an emphasis on postnatal inflammation have been reported (5). Antenatal endotoxins can induce lung inflammation, leading to high neonatal mortality and subsequent arrests in alveolarization in the lungs of infant rats, which is one of the classic animal models of BPD (6). Lung inflammation is recognized as an important risk factor for predisposing the infant to BPD before birth, and cytokines, including interleukin (IL)-1β and interferon (IFN)-γ, play an important roles in this process (4, 7). IFN-γ is a proinflammatory cytokine detected in lungs exposed to inflammation and an inducer of effector mechanisms, thereby qualifying it as an early mediator of BPD development (6, 8). Recognizing that inflammatory cytokines are involved in lung injury, various investigators explored the potential of inflammatory cytokines as biomarkers for BPD in premature infants (9). Higher serum concentrations of IL-1β and IFN-γ and lower concentrations of other cytokines (IL-17) are associated with the development of BPD in preterm babies (10).

Vitamin D (VitD) is involved in many biological processes in addition to its role in the regulation of bone and calcium homeostasis (11). VitD can be metabolized into steroid-hormone metabolites, including 1, 25-dihydroxyvitamin D_3_ [1,25(OH)_2_ D_3_] (12). Since the discovery of VitD receptors (VDRs) in a variety of cells of the adaptive immune system, such as B and T lymphocytes (13), it has appeared conceivable that VitD might modulate the immune system. Epidemiological studies suggested a relationship between VitD with the incidence of autoimmune diseases (14, 15). Additionally, VitD supplementation prevents both the initiation and progression of experimental autoimmune encephalomyelitis and experimental models of multiple sclerosis and rheumatoid arthritis (16, 17). Recently, VitD deficiency or insufficiency was associated with respiratory disease in children, as revealed by accumulating epidemiologic evidence (18–20). Furthermore, Fettah et al. (21) reported that lower cord-blood 25(OH)D levels might be associated with increased risk of respiratory distress syndrome in preterm infants. However, the potential mechanisms of VitD-specific regulation of proinflammatory factors remain incompletely understood.

In this study, we reported that VitD supplementation relieved alveolar development impaired by lipopolysaccharide (LPS) by decreasing the production of inflammatory factors, such as IFN-γ, in a BPD rat model.

## Materials and Methods

### Experimental Animals

Timed-pregnancy Sprague-Dawley rats were provided by the Shanghai Laboratory Animal Center (Shanghai, China) and the experimental protocol was approved by the ethics committee of Xinhua Hospital (Shanghai, China).

### Animal Model and Study Design

We used an established animal model of BPD that was described previously (22, 23). Briefly, at 16.5 days of gestation (term: 22 days), pregnant rats were prepared for receiving intra-amniotic injections. The pregnant rats were randomly assigned to the saline control group, LPS group, or LPS with VitD group. The saline control group received 5 µL of normal saline per amniotic sac, the LPS group received 1 µg of LPS (*Escherichia coli* 055:B5; Sigma-Aldrich, St. Louis, MO, USA) diluted to 5 µL with normal saline per amniotic sac, and the LPS with VitD group received 1 µg of LPS and 5 pg of 1, 25(OH)_2_D_3_ (Sigma-Aldrich) diluted to 5 µL with normal saline per amniotic sac. The day when the pups were born was counted as postnatal day 0 (P0). The pups from the LPS with VitD group were injected with 1, 25(OH)_2_D_3_ (1 ng/g) according to their weight daily from P0 to P7, whereas pups from the saline control and LPS groups were injected with normal saline at the same volume.

### Lung and Spleen Processing

At P1, P3, and P7, 20–30 newborn rats from each model or control group were anesthetized by intraperitoneal injection of 5% chloral hydrate, and whole lungs and spleen were aseptically collected by a chest- and abdomen-opening procedure. The right bronchus and trachea were ligated, and spleen tissues were decollated from the great bend of the stomach. The pups then received intratracheal instillation of buffered formaldehyde [4% paraformaldehyde solubilized in phosphate-buffered saline (PBS); pH 7.4] at a pressure of 20 cm H_2_O for 20 min. For histological analysis, the left upper lobes were formaldehyde-fixed and paraffin-embedded. Serial 5-mm thick sections were stained with hematoxylin and eosin (H&E). For IFN-γ and IL-4 measurements, the right lung lobes without perfusion and spleen tissues were excised, frozen in liquid nitrogen, and stored at −80°C.

### Lung Morphometry

Three pups were selected from each group, and five random non-overlapping fields in one distal lung section per pup were utilized for morphometric examinations. The terminal airspace, secondary septa, and myofibroblasts in each field were manually counted. The mean linear intercept (MLI) was determined by superimposing a predetermined grid with set randomly placed lines totaling 1 mm in actual length onto the image and counting the number of times the lines cross an air–tissue interface. The actual MLI was calculated as the inverse of the number of air–tissue interfaces per millimeter (1,000 µm) and was used to estimate the mean distal airspace size.

### IFN-γ and IL-4 Measurements by Real-time Polymerase Chain Reaction (PCR)

Total mRNA was extracted from five to seven right upper lung lobe and spleen tissues from all groups at P1, P3, and P7. The samples were subjected to reverse transcription and real-time PCR (Life Technologies, Carlsbad, CA, USA) according to manufacturer instructions. Real-time PCR was performed using SYBR Green PCR master mix (Applied Biosystems), and data were collected and quantitatively analyzed on an ABI Prism 7500 sequence detection system (Applied Biosystems). All primers were obtained as amplimer sets from Sangon Biotech (Shanghai, China). The following primers were used: β-actin (forward, 5′-GGAAATCGTGCGTGACATTA-3′; reverse, 5′-AGGAAGGAAGGCTGGAAGAG-3′); IFN-γ (forward, 5′-AGGTGAACAACCCACAGAT-3′; reverse, 5′-CTTCTTATTGGCACACTCTCTAC-3′); and IL-4 (forward, 5′-ACAAGGAACACCACGGAGAA-3′; reverse, 5′-CAGACCGCTGA- CACCTCTACA-3′). Model data were standardized to β-actin.

### IFN-γ and IL-4 Enzyme-Linked Immunosorbent Assay (ELISA)

The production of IFN-γ and IL-4 in the five to seven right lower lung lobe and spleen tissues from each group at P1, P3, and P7 were assessed using the IFN-γ and IL-4 ELISA kit (R&D Systems, Minneapolis, MN, USA) according to manufacturer instructions. Tissues homogenates were prepared in 1× PBS and frozen at −80°C. Homogenates were subjected to two cycles of thawing and freezing, centrifuged, and supernatants were collected for ELISA measurement. Total protein in tissues was determined using the Pierce BCA protein assay kit (Beyotime Biotechnology, Shanghai, China) and used for normalization purposes.

### Bisulfite Sequencing PCR

The genomic DNA from four lung tissues collected at P3 from each group was extracted using a blood/cell/tissue genomic DNA extraction kit (Tiangen Biotech, Beijing, China). After elution with preheated 70°C sterile water, the DNA was collected. Thereafter, DNA specimens were treated with a DNA methylation kit (Millipore, Billerica, MA, USA) and amplified. PCR amplification conditions were 95°C for 5 min, 95°C for 10 s, 50°C for 20 s, 72°C for 30 s for a total of 35 cycles, followed by one cycle at 4°C for 5 min. PCR primers were designed using MethPrimer software (http://www.urogene.org/methprimer/) and included forward primer (5′-AATAAATGTTTATTGTGTTGTATTTTG-3′) and reverse primer (5′-AA-CTCCTATATTAAATCAAAAAATCC-3′). The amplicons were confirmed by agarose gel electrophoresis and sequenced using a BiQ Analyzer (24).

### Preparation, Culture, and Treatments of Bone Marrow-Derived Macrophages (BMDMs)

Bone marrow-derived macrophages were isolated from the marrow of femurs and tibias from 4-week-old male rats (100–150 g). Cells were then differentiated into BMDMs in Roswell Park Memorial Institute medium containing 10 ng/mL recombinant M-CSF (Sigma-Aldrich), 10% fetal bovine serum (Gibco, Paisley, UK), and streptomycin/penicillin (Gibco). Cell-culture population consisted of nearly 80% BMDMs at 7 days after seeding. The cells were harvested, washed with complete media, and plated in 6-well plates with or without 5-Aza-2′-deoxycytidine (5-aZac; 10 µmol/L; Abcam, Cambridge, UK) for 48 h. The cells without 5-aZac were stimulated with PBS, LPS (1 µg/mL), or LPS (1 µg/mL) + 1, 25(OH)_2_D_3_ (100 pg/mL) for 12 h, respectively, whereas cells with 5-aZac were stimulated with LPS (1 µg/mL) + 1, 25(OH)_2_D_3_ (100 pg/mL) for 12 h. After storage in liquid nitrogen, the cells and supernatant of five samples from four groups were transferred to a −80°C freezer, respectively, and then subsequently used for real-time PCR and ELISA analyses.

### Flow Cytometry

Spleen tissues from three pups from each group were collected at P15, and single-cell suspensions were prepared by homogenization through a 75-µm cell strainer. Red blood cells were lysed (RBC lysing buffer; Beyotime Biotechnologies), whereas splenocytes were washed and resuspended in FACS buffer (Hank’s Balanced Salt Solution supplemented with 2% fetal calf serum). The cells were characterized by surface staining with anti-rat antibodies against CD3 and CD8. For intracellular staining, surface-stained cells were washed in FACS buffer and permeabilized with IC fixation buffer/permeabilization buffer for 30 min, followed by washing and staining in FACS buffer with anti-rat antibodies against IFN-γ for 30 min. All antibodies were purchased from eBioscience (San Diego, CA, USA), and all flow-cytometry experiments were performed using LSR II (BD Systems, UK).

### Cohort Study Design and Participants

This was a prospective birth cohort study that recruited 1,071 maternal–child pairs between 2012 and 2013 at Xinhua Hospital and the International Peace Maternity and Child Hospital, two large tertiary hospitals in Shanghai, China. Prior to delivery, written informed consent was obtained from the mothers and trained nurses conducted face-to-face interviews (25, 26). At birth, study nurses collected anthropometric details and umbilical-cord blood from the newborns, when available. Ethics approval was obtained by the Ethics Committees of both Xinhua Hospital and the International Maternal and Children Care Hospital. Because the mode of delivery might affect the inflammation status of the neonate, we only included data obtained from cesarean sections on maternal request. Mothers diagnosed with gestational complications were excluded. In the final analysis, 86 mother–infant pairs were included.

### Measurement of VitD, INF-γ, and IL-4 in Maternal and Umbilical Cord Blood

We used liquid chromatography tandem mass spectrometry (LC-MS/MS) to detect serum 25(OH)D according to the procedure reported by our previous study (25). INF-γ and IL-4 were measured using an ELISA kit (LDN GmbH & Co., Nordhorn, Germany) according to manufacturer protocol.

### Statistical Analysis

Data are presented as the mean ± SD. The variance test was equal. The Student’s *t*-test and one-way ANOVA analysis were performed using SPSS 18.0 software (SPSS, Inc., Chicago, IL, USA) to analyze the statistical significance of the comparisons. The Student–Newman–Keuls method was used to compare differences of IFN-γ and IL-4 between VitD groups. Statistical significance was defined as a *P* < 0.05. VitD levels <20 ng/mL were generally considered as deficiencies. We stratified all samples into three groups (<20, 20–30, and ≥30 ng/mL). The difference in VitD level between cord blood and that obtained mid-trimester was calculated as the change in VitD as was stratified into three groups (<0, 0–10, and ≥10 ng/mL).

## Results

### VitD Treatment Improves Alveolar Development in Antenatal LPS-treated Rats

First, we tested whether VitD could rescue alveolar development following LPS exposure. No fetal death occurred in the control group, and almost all rats survived until the end of the experiment. The survival rates of the pups in the LPS group and the pups in the LPS with VitD group were similar (80 and 83.5%, respectively). The lung structures of the pups in the LPS group had typical characteristics of alveolar simplification, as indicated by enlarged alveoli with a decreased terminal airspace, decreased secondary septa, and an increased MLI.

**Figure 1:**
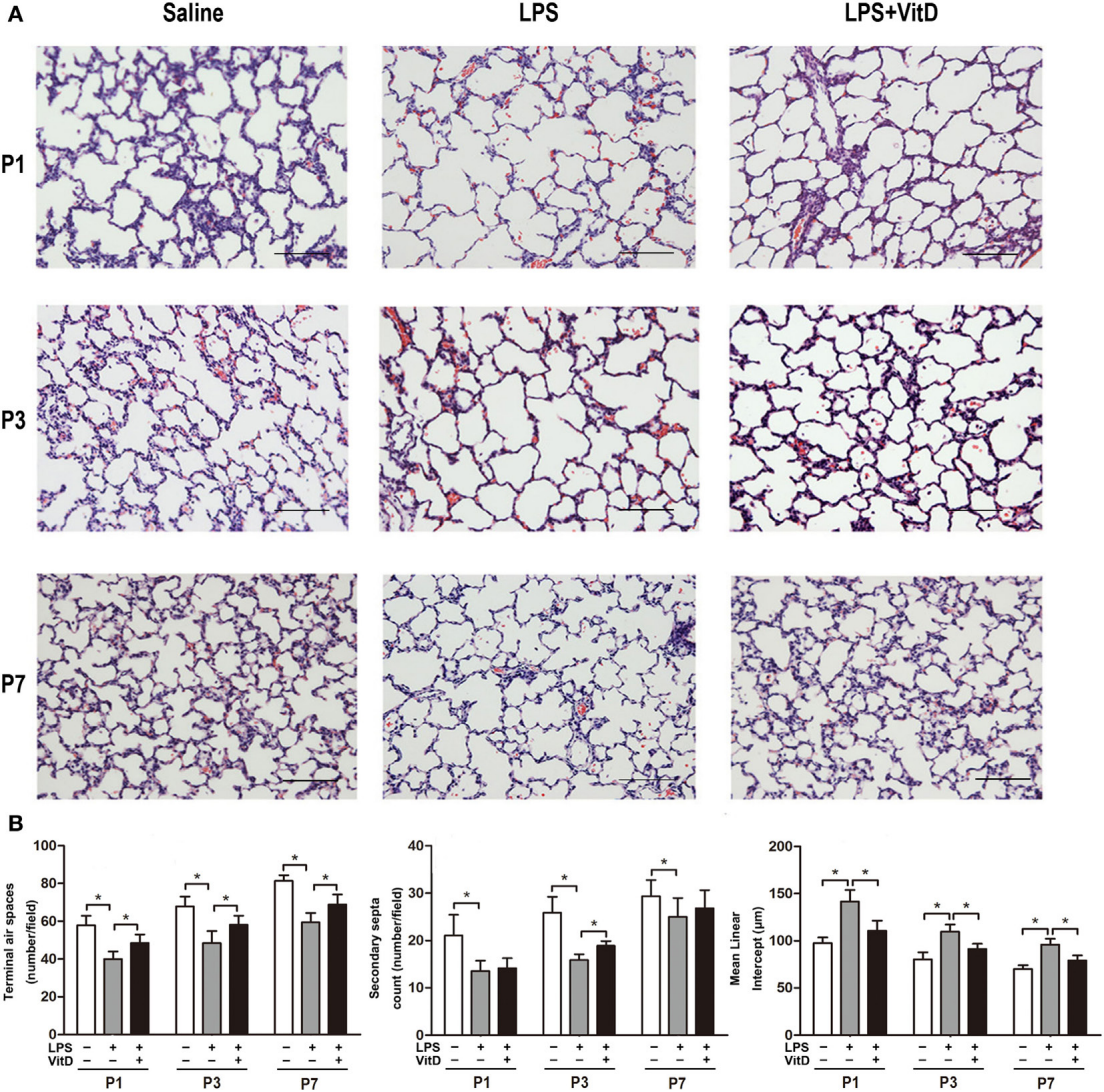
Vitamin D treatment improves lung structure in antenatal lipopolysaccharide (LPS)-treated rats. **(A)** Representative lung sections stained with H&E. **(B)** Quantification of the terminal airspace, secondary septa, and mean linear intercept. Bars, 200 µm. Data represent the mean ± SD. *p < 0.05 (*n* = 3 pups per group, with at least five replicates).

We then examined the effect of VitD treatment on LPS-induced alveolar simplification (Figure 1A). Pups receiving LPS and VitD co-treatment showed a significantly increased terminal airspace and significantly reduced MLI at P1, P3, and P7 as compared with pups receiving LPS treatment alone (*P* < 0.05). In terms of the secondary septa, the difference between P1 and P7 was not significant in the LPS groups with or without VitD (Figure 1B). These results suggested that VitD effectively alleviated lung alveolar structure simplification induced by LPS.

### IFN-γ mRNA and Protein Levels in Antenatal LPS-Treated Rats Are Regulated by VitD

**Figure 2:**
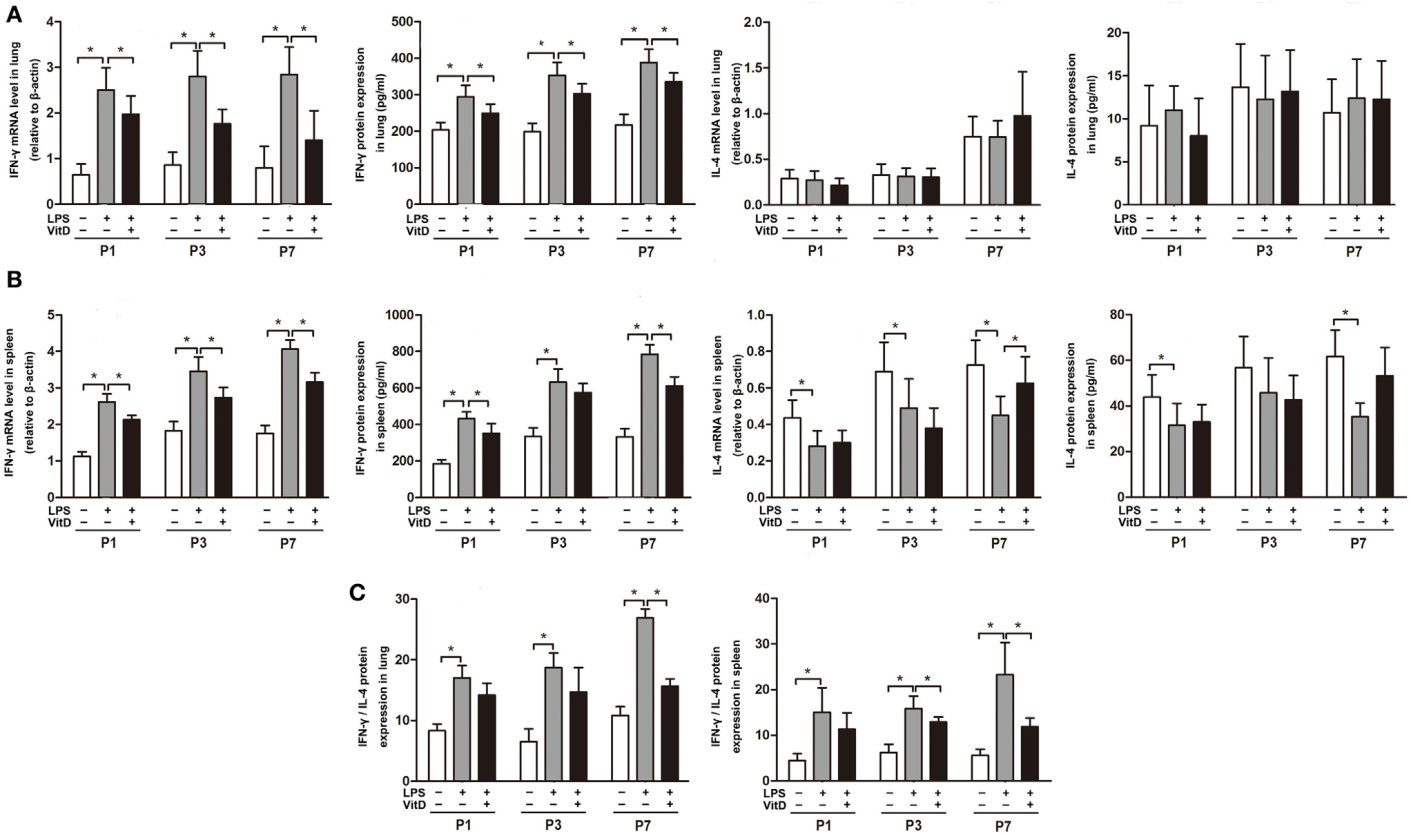
Interferon (IFN-γ) mRNA and protein levels in antenatal lipopolysaccharide (LPS)-treated rats are regulated by VitD. **(A)** IFN-γ and interleukin (IL)-4 mRNA and protein levels in rat lung tissue and **(B)** spleen. **(C)** IFN-γ/IL-4 ratio in the lung and spleen. Data represent the mean ± SD. *p < 0.05 (*n* = 5–7 pups for each group; experiments were repeated five times for each data point).

We then investigated the effects of VitD treatment on the regulation of inflammation. VitD suppressed LPS-induced IFN-γ mRNA and protein expression in the lung at P1, P3, and P7 (*P* < 0.05) (Figure 2A). Additionally, IFN-γ mRNA and protein expression in the spleen were similarly significantly decreased by VitD at P1 and P7 as compared with pups in the LPS group (*P* < 0.05) (Figure 2B), whereas levels of IL-4 in lung and spleen tissues were insuscepti ble to VitD. We also contrasted the IFN-γ/IL-4 protein-expression ratio in the lung and spleen, finding that antenatal LPS injection increased the IFN-γ/IL-4 ratio in lung and spleen tissues, whereas VitD treatment significantly decreased this ratio in lung tissue at P7 and in spleen tissue at P3 and P7 (Figure 2C).

### VitD Supplementation Changes the Percentage of IFN-γ-Producing CD8+ T Cells in the Spleen of BPD Rats

**Figure 3:**
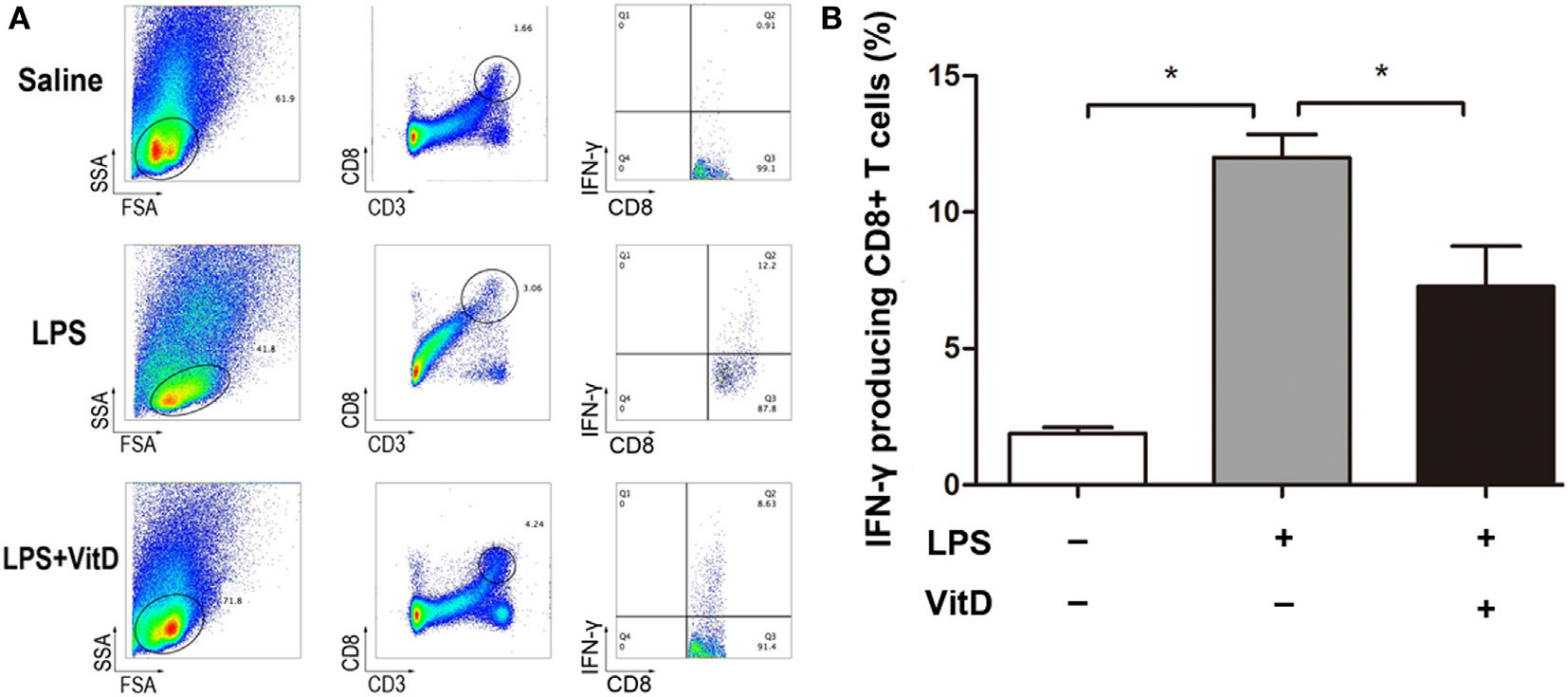
VitD supplementation changes the percentage of interferon (IFN)-γ-producing CD8+ T cells in the spleen of bronchopulmonary dysplasia rats. **(A)** A representative flow plot of IFN-γ-producing CD8+ T cells in each group. **(B)** Quantitative analysis of the percentage of IFN-γ intracellular-stained CD8+ T cells. Data represent the mean ± SD. *p < 0.05 (*n* = 3 pups for each group; each experiment was repeated at least four times).

Our attempt to examine whether VitD treatment impacted the numbers of CD4+ T cells in lung and spleen tissues revealed that only a tiny minority of CD4+ T cells were detected in these tissues. Because IFN-γ can also be expressed in CD8+ T cells, we investigated whether VitD treatment was associated with a reduction in the numbers of IFN-γ-producing CD8+ T cells. In lung tissues, CD8+ T cells were not randomly dispersed, allowing their accurate detection; however, comparison of the number of IFN-γ-producing CD8+ T cells in spleen tissue from BPD rats in the LPS and LPS with VitD groups at P15 revealed that the VitD treatment led to a significant decrease in the frequency of IFN-γ-producing CD8+ T cells in the spleen, as indicated by a reduction in the potential for IFN-γ production (Figure 3A, B).

### VitD Treatment Alters the Methylation Status of the VitD-Response Element (VDRE) in the IFN-γ-Promoter Region

**Figure 4:**
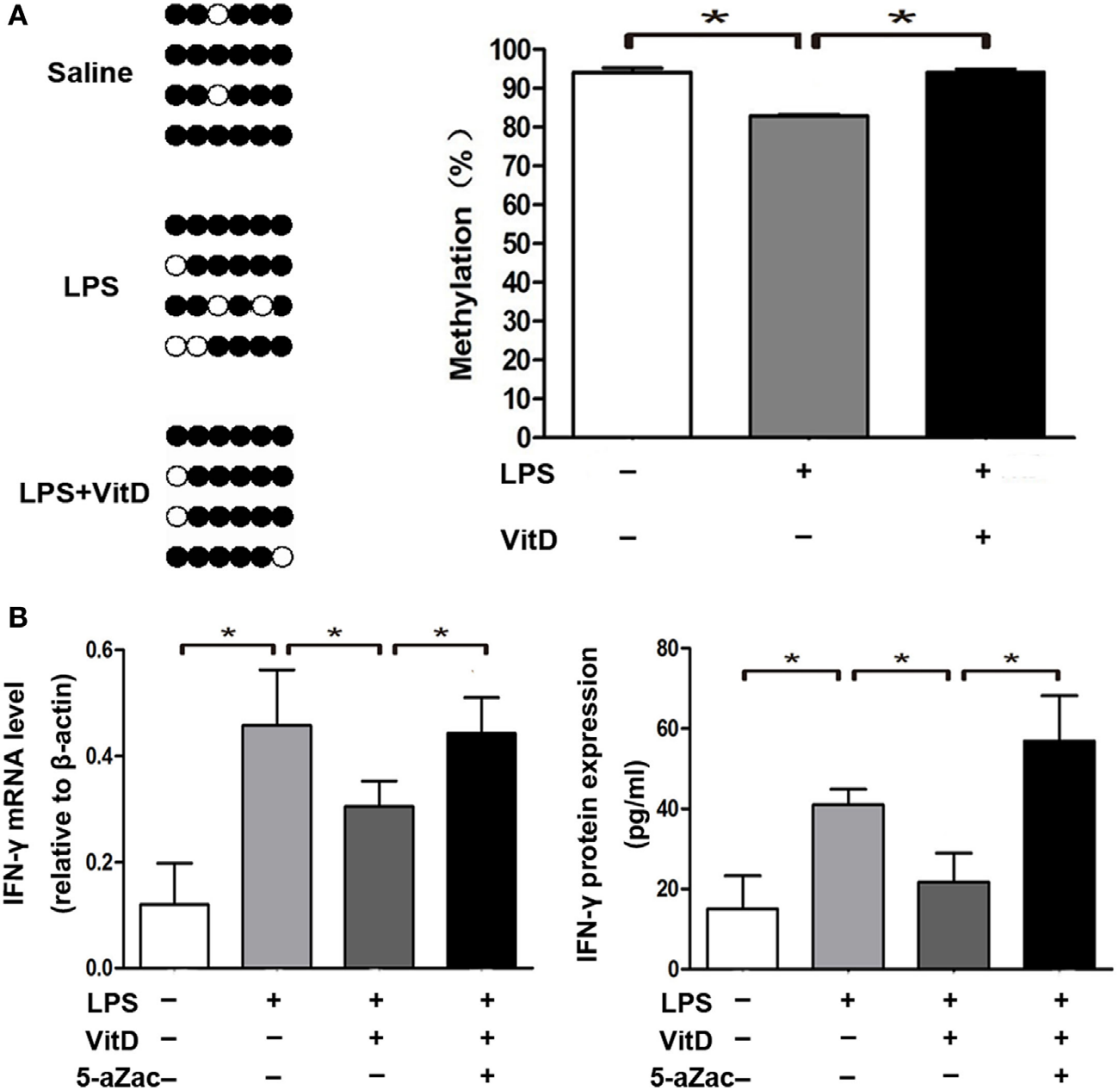
Vitamin D treatment alters the methylation status of the VitD-response element in the interferon (IFN)-γ-promoter region. **(A)** DNA methylation in the IFN-γ-promoter region in lung tissue from all groups. Results showing representative methylation and quantification of the proportion of methylation in the three experimental groups are shown (*n* = 4 pups at P3 for every group). **(B)** IFN-γ mRNA and protein levels in macrophages after 48-h treatment with the inhibitor 5-aZac (10 µmol/L) (*n* = 5 samples from the four groups; each experiment was repeated four times). Data represent the mean ± SD. *p < 0.05.

We then analyzed the relationship between the percentage of methylation of the VDRE in the IFN-γ-promoter region combined with those of the VDR and VitD levels in the lung tissue of pups from all of the groups at P3. There are six sites in the IFN-γ-promoter region (−108 to +64 bp) that were combined with VDR. We observed that lung-tissue samples of rats from all groups showed highly methylated and that VitD treatment significantly increased the methylation percentage of the VDRE in the IFN-γ-promoter region as compared with LPS treatment (*P* < 0.05) (Figure 4A). Furthermore, the regulation of IFN-γ expression by VitD treatment disappeared following administration of the methylation inhibitor 5-aZac (Figure 4B).

### VitD Regulates IFN-γ and IL-4 Levels in Cord Blood

**Figure 5:**
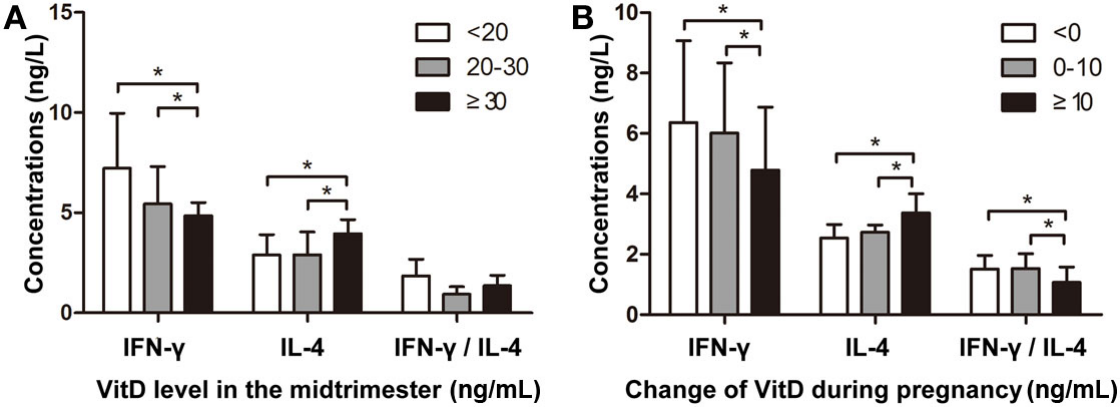
Vitamin D regulates interferon (IFN)-γ and interleukin (IL)-4 levels in cord blood. **(A)** The association between VitD concentration during the mid-trimester and IFN-γ and IL-4 levels in cord blood. The *Y*-axis represents IFN-γ and IL-4 concentrations and the IFN-γ/IL-4 ratio [VitD levels: <20 ng/mL (*n* = 36); 20–30 ng/mL (*n* = 32); ≥30 ng/mL (*n* = 18)]. **(B)** The association between altered VitD supplementation during pregnancy and IFN-γ and IL-4 levels in cord blood. The *Y*-axis represents IFN-γ and IL-4 concentrations and IFN-γ/IL-4 ratio [changes in VitD concentration: <0 ng/mL (*n* = 33); 0–10 ng/mL (*n* = 29); ≥10 ng/mL (*n* = 24)]. Data represent the mean ± SD. *p < 0.05.

To confirm our findings, we assessed correlations between maternal VitD exposure and IFN-γ and IL-4 levels in newborns. The median maternal serum VitD concentration at 24–28 gestational weeks was 20.1 ng/mL (10.2–26.8 ng/mL). Additionally, neonatal IFN-γ levels were significantly lower in the group of pregnant mothers with VitD concentrations ≥30 ng/mL, whereas IL-4 levels were significantly higher in this group (Figure 5A). Moreover, increased VitD intake during the last trimester resulted in lower neonatal INF-γ levels and INF-γ/IL-4 ratios in cord blood (Figure 5B). This trend was similar to those observed in the animal model.

## Discussion

Bronchopulmonary dysplasia pathogenesis is multifactorial; however, all of its triggers cause pulmonary inflammation and might, therefore, inhibit lung morphogenesis. Inflammation is generally believed to be a primary mediator of injury associated with BPD pathogenesis (27, 28). The central role of perinatal inflammation and its possible consequence of fetal inflammatory response syndrome led the BPD Group of the American Academy of Pediatrics to make the identification of anti-inflammatory agents associated with the development of perinatal inflammation a research priority (28).

Vitamin D supports an emerging hypothesis that early maternal VitD deficiency might contribute to a high risk for subsequent asthma and BPD during infancy (18, 29, 30). Previous studies also suggest that VitD treatment might be protective against respiratory distress syndrome in preterm neonates (12). In our study, we verified that sustainable VitD supplementation during the fetal and neonatal periods could indeed alleviate morphological changes of the lung in LPS-induced BPD animal models.

Interferon -γ induced by LPS is mainly involved in cellular immunity and reportedly plays an important role in the development of BPD (31). In the current study, we observed that IFN-γ expression was decreased along with VitD supplementation. Evidence suggests that VitD might stimulate T cell differentiation and suppress inflammatory responses to environmental exposures (32). This results in a variety of important biological activities, as not only is IFN-γ a vital initiator of inflammation by LPS, it also promotes T helper (Th)1 cell responses and results in Th1 and Th2 responses to environmental exposure, thereby conferring antimicrobial activity (32, 33). Th1 cells secrete proinflammatory factors (primarily IFN-γ) and are mainly involved in cellular immunity. Whereas Th2 cells produce anti-inflammatory factors (primarily IL-4) and are associated with humoral immunity. The levels of IFN-γ and IL-4 can indirectly reflect situations associated with Th1/Th2 shifting and the balance between anti-inflammatory and proinflammatory responses (34). Here, we also detected changes in IL-4 levels and compared IFN-γ/IL-4 ratios in the treatment groups. These results showed that VitD mainly decreased the IFN-γ expression, thereby promoting an imbalance of IFN-γ and IL-4 in BPD rats, leading to suppression of local inflammation in lung tissue and of systemic inflammation. Neonatal T cell responses were historically thought to be deficient due to immaturity (8), which might explain why CD4+ T cells and portions of CD8+ T cells were undetectable in our research. Our data suggested that VitD treatment was associated with reductions in IFN-γ-producing CD8+ T cells in spleen tissue. Moreover, these results indicated a potential mechanism associated with VitD-related improvement of lung structure in LPS-induced BPD rats through its regulation of IFN-γ expression.

However, the underlying mechanisms associated with the regulation of IFN-γ expression by VitD remain unclear. VitD can regulate gene expression and exert widespread immunomodulatory effects (35–37). In the present study, we explored associations between VitD and IFN-γ expression by analyzing the relationship between methylation percentages in the VDRE located in the IFN-γ-promoter region. As we hypothesized, VitD increased the percentage of methylation of VDRE in the IFN-γ-promoter region to a greater extent than that of LPS treatment alone. Recent studies showed that DNA methylation, a key epigenetic mechanism, is altered in children exposed to air pollutants and environmental tobacco smoke early in life (38). An example of this is the intrauterine or postpartum environment, which causes DNA methylation and makes infants susceptible to chronic obstructive pulmonary disease, asthma, and other respiratory system diseases (39). Previous studies indicated that epigenetic mechanisms play a significant role in the inflammatory process, regardless of whether it occurs locally or systemically. Additionally, altered DNA methylation in lung tissues plays a major role in LPS-induced lung injury (40). Interestingly, 1,25(OH)_2_D_3_ can enter cells and bind to receptors, during which the resulting complex heterodimerizes with the retinoid X receptor and binds to the VDRE in the promoters of target genes of pro-inflammation factors, thereby affecting their transcription (41). The VDR protein physically interacts with co-activator and co-repressor proteins, which, in turn, come into contact with chromatin modifiers, such as histone acetyltransferases and histone methyltransferases, and chromatin remodelers (11). Based on these findings, we assessed the six CpG sites in the VDRE of the IFN-γ-promoter region, which are mainly regulated by VitD. However, we acknowledge the possibility of missing other CpG sites in the promoter region; therefore, further studies are needs to understand other molecular mechanisms underlying epigenetic regulation of IFN-γ expression by VitD. To verify our hypothesis concerning the regulation of methylation by VitD, we observed that administration of the nonspecific methylation inhibitor 5-aZac significantly blocked VitD regulation of IFN-γ expression in rat BMDMs. These results suggested that VitD mediated the DNA methylation associated with downregulation of IFN-γ levels during LPS-induced BPD.

Vitamin D is hypothesized as being important to immune and inflammatory responses (42); however, it is unknown whether VitD suppresses proinflammatory cytokine activation *in vivo*, particularly in the context of fetal immune ontogeny. In this study, we found that neonatal INF-γ levels were significantly lower in the group of mothers with VitD concentrations ≥30 ng/mL during the mid-trimester, whereas IL-4 levels were significantly higher in this group. These results might suggest that the higher VitD concentration was associated with altered INF-γ levels to suppress inflammation. Additionally, increased intake of VitD during the last trimester resulted in lower INF-γ levels and INF-γ/IL-4 ratios in cord blood, a trend similar to that observed in the animal model. These findings also supported our hypothesis that VitD supplementation could relieve inflammatory injury through the suppression of IFN-γ production. Furthermore, although maternal VitD exposure during pregnancy was associated with neonatal IFN-γ levels, additional intervention studies are necessary to prove causality.

In summary, our results established a new molecular mechanism pertaining to the VitD-related improvement of lung morphogenesis following intrauterine inflammation. Our findings showed that VitD alleviated morphological changes of the lung in LPS-induced BPD rats. Most importantly, we demonstrated that VitD supplementation suppressed IFN-γ production and inhibited the inflammatory response.

## Ethics Statement

Timed-pregnancy Sprague-Dawley rats were provided by the Shanghai Laboratory Animal Center and the experimental protocol was approved by the ethics committee of Xinhua Hospital. Ethics approval of the prospective birth cohort study was obtained by the Ethics Committees of both Xinhua Hospital and the International Maternal and Children Care Hospital.

## Author Contributions

All authors contributed to the intellectual content of the manuscript and approved the manuscript version submitted for publication. CL, ZC, WL, and LH performed the experiments; CL, ZC, and LH analyzed the data and prepared the figures; CL, ZC, LH, and YZ interpreted the experimental results; CL drafted the manuscript; YZ was responsible for the conception and design of the research and approved the final version of the manuscript; LH and YZ edited and revised the manuscript.

## Conflict of Interest Statement

The authors declare that the research was conducted in the absence of any commercial or financial relationships that could be construed as a potential conflict of interest. The reviewer LD and handling editor declared their shared affiliation.
